# A Comprehensive Overview of Technologies for Species and Habitat Monitoring and Conservation

**DOI:** 10.1093/biosci/biab073

**Published:** 2021-07-28

**Authors:** José J Lahoz-Monfort, Michael J L Magrath

**Affiliations:** School of Ecosystem and Forest Sciences, University of Melbourne, Melbourne, Victoria, Australia; Wildlife Conservation and Science, Zoos Victoria and with the School of BioSciences, University of Melbourne, Melbourne, Victoria, Australia

**Keywords:** sensors, wildlife monitoring, biologging, telemetry, computing

## Abstract

The range of technologies currently used in biodiversity conservation is staggering, with innovative uses often adopted from other disciplines and being trialed in the field. We provide the first comprehensive overview of the current (2020) landscape of conservation technology, encompassing technologies for monitoring wildlife and habitats, as well as for on-the-ground conservation management (e.g., fighting illegal activities). We cover both established technologies (routinely deployed in conservation, backed by substantial field experience and scientific literature) and novel technologies or technology applications (typically at trial stage, only recently used in conservation), providing examples of conservation applications for both types. We describe technologies that deploy sensors that are fixed or portable, attached to vehicles (terrestrial, aquatic, or airborne) or to animals (biologging), complemented with a section on wildlife tracking. The last two sections cover actuators and computing (including web platforms, algorithms, and artificial intelligence).

For decades, technology has played an important role in how we study habitats and species of conservation concern, as well as helping us deal with threats to biodiversity. From humble beginnings of handcrafted devices, some technologies (e.g., camera trapping, radio tracking) have become standard tools in wildlife studies. Furthermore, the last couple of decades have seen an unprecedented explosion in technological development at all levels of society, including the digital revolution brought by computing and near-global connectivity, the rise of the DIY or maker communities, and more flexible manufacturing associated to the Fourth Industrial Revolution (Berger-Tal and Lahoz-Monfort 2018). With cheaper and faster than ever technology prototyping and manufacturing, novel ways of developing technology are emerging, including collaborative open source. All these developments have been slowly trickling into conservation, with a very broad range of established and emerging technologies developed, adopted from other disciplines, or used in novel ways and being trialed in the field (Pimm et al. 2015). Finally, there is a recent and growing international push for the conservation community to become innovation leaders rather than users of technologies developed for other purposes, progressively leading to the development and awareness of “conservation technology” as a discipline (Joppa 2015, Berger-Tal and Lahoz-Monfort 2018, Lahoz-Monfort et al. 2019). Despite all the exciting uses and developments, there has been no comprehensive overview of the use of this broad range of technologies for conservation purposes. We aim to provide the first snapshot of the current landscape of this emerging discipline as of 2020. It offers an up-to-date entry point for those coming into conservation (e.g., graduate students, new practitioners) and a broad but comprehensive overview of existing possibilities for those wanting to expand their use of technology or scout for new options. This overview will be equally useful for people working in other areas of applied ecology (e.g., wildlife monitoring for management of harvested populations) as most of these technologies will be useful beyond conservation.

## Conservation technology

Although the term *technology* can include practically any expression of human ingenuity applied to solving practical problems, in the present article, we follow a commonly used more restrictive definition as “machinery and equipment developed from the application of scientific knowledge” (Lexico 2021), including both physical tools (devices and machines, often relying on electronics) and more abstract methods associated with them (including computer programs and algorithms). Conservation technology naturally extends this definition to technology that is useful to achieving biodiversity conservation goals. We focus our overview on technologies that play a specific role (i.e., excluding generic devices such as laptops), whether they have been specifically created with biodiversity in mind (e.g., radio collar) or not (e.g., drone). We include two categories. First, *established* technologies are routinely deployed in conservation, normally backed by substantial field application and a solid body of scientific literature. This does not imply old fashioned or underperforming: Most have continued improving in functionality and performance over time. Second, *novel* technologies (or, often, novel uses of existing technologies) on the other hand would typically be at trial stage and often considered a risky investment in a critical conservation intervention. The conservation community at large would often view them with interest, and some programs and institutions would decide to invest in them. In covering novel applications of technology and giving them profile, we hope to hasten the understanding, adoption and acceptance of the possibilities brought by the latest options. Because the primary literature is necessarily limited, we have provided links to websites when possible. The line between established and novel is obviously fuzzy, and established technologies (e.g., acoustic loggers) can be used in novel ways (e.g., deployed from a drone over forest canopy). For well-documented established technologies, we provide references to more detailed discussions or specific reviews; we focus in the present article on their latest developments and mention future opportunities for improvement if relevant.

We have excluded from this overview a third category, *emerging* technologies, whose potential to contribute to conservation may have been claimed but not yet demonstrated in practice. These include both existing technologies with still largely untapped potential for application in conservation and technologies at conceptual or early stages of development. The former category includes some sensing technologies (the Internet of Things, mobile crowdsensing), 3D printing and additive manufacturing, and some computing technologies (virtual reality and augmented reality, gamification, blockchain, culturomics, and i-ecology). The latter includes advanced robotics, smart dust, synthetic biology (including gene drive), and biobatteries. We consider these beyond the scope of this already broad article because the purpose of covering emerging technologies would be different, focusing on opportunities rather than existing applications, therefore being somewhat speculative. Again, there is some degree of subjectivity in classifying a technology as *emerging* rather than *novel*.

Finally, we considered biotechnology (based on the processing of biological samples) outside the scope of this overview, focusing instead on technologies that are based on physical phenomena (e.g., sound or electromagnetic waves, including light and heat). We acknowledge the important contribution to conservation that some of these have, including conservation genomics, metagenomics (Russello et al. 2020) and genetic barcoding (Hebert et al. 2003), environmental DNA (Beng and Corlett 2020), and stable isotopes (Rubenstein and Hobson 2004). Beyond these specific references, the application of biotechnology to conservation was recently reviewed (Corlett 2017).

We acknowledge that the successful contribution of technology to conservation hinges on many important (often critical) considerations that are not purely technical. These include, for example, challenges associated with field deployment, ethical and social implications of the use of technology, hype or inflated expectations, overreliance on technological solutions. These deserve their own study and are not discussed in the present article, which is focused on the development and use of technology for conservation purposes. We have also purposely excluded any technology specific to animal husbandry and veterinary science (e.g., surgery or reproductive technologies).

**Figure 1.**
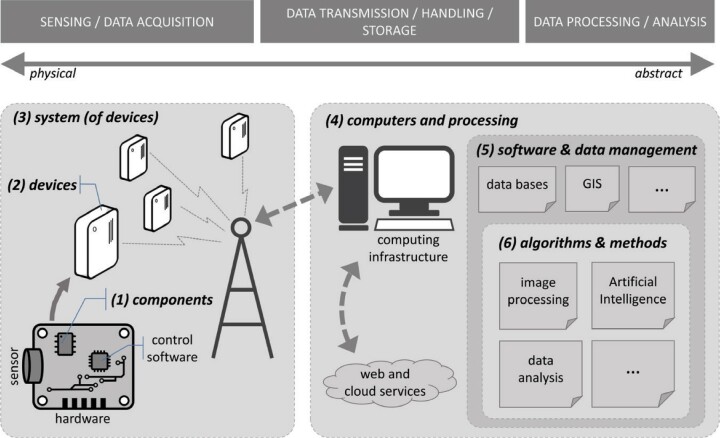
Illustrative example of a technology pipeline (from sensing to analysis) and associated types of technologies, including (1) components, (2) devices, (3) systems, (4) computers and processing, (5) software and data management, (6) algorithms and methods. The example represents a simplified view of a network of fixed sensors that transmit data in real time to a back-end computing system.

An overview of a broad discipline such as conservation technology can be structured following different criteria (e.g., by conservation objective). We have chosen to follow what we believe is a natural description of the technology pipeline (figure 1), which goes from the physical (electronic components) to the abstract (algorithms): sensing and data acquisition (mostly hardware dependent); data transmission, handling, and storage (hardware but mostly computing and software); and data processing, analysis, and use (largely relying on software and algorithms).

**Figure 2.**
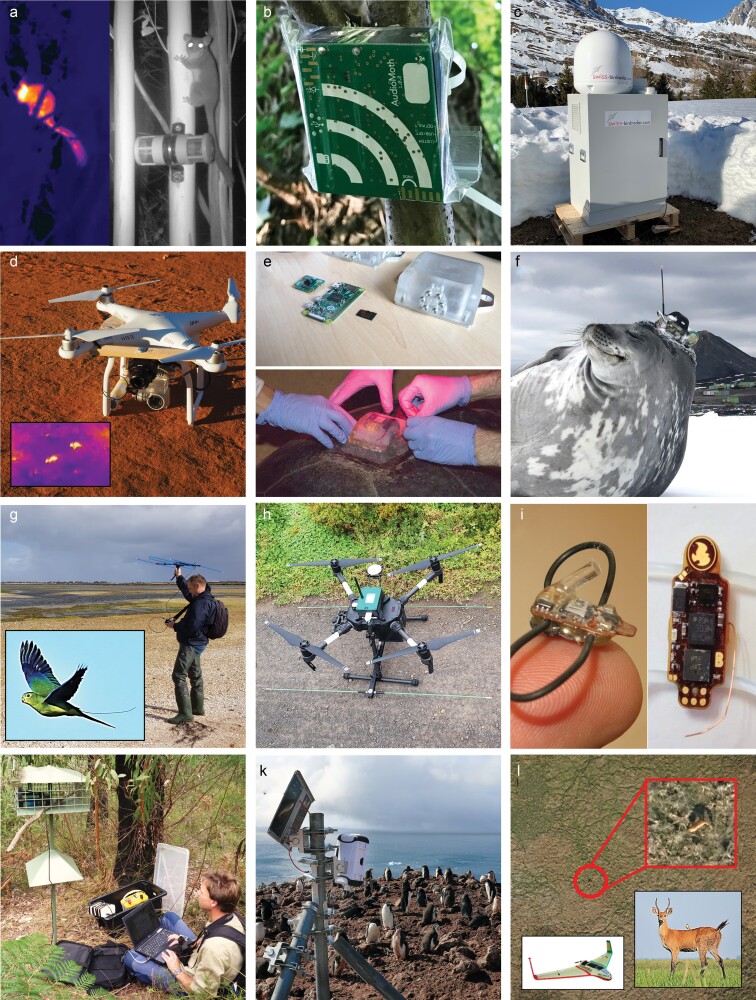
Illustrative examples of conservation technologies. (a) Thermal and camera trap images (night infrared illumination) of Leadbeater's possums. Photograph: Zoos Victoria. (b) Low-cost open-source acoustic logger (AudioMoth) for passive acoustic monitoring. Photograph: Open Acoustic Devices. (c) Small vertical-looking pulse radar (BirdScan) used to study bird migration. Photograph: Swiss Ornithological Institute. (d) Small multirotor UAV (DJI Phantom3) with color camera and thermal sensor (FLIR Vue Pro R) used to detect wildlife (inset: two bridled nail-tail wallabies). Photographs: José Lahoz-Monfort. (e) Raspberry Pi–based video logger for sea turtles (Arribada PS-C). Photograph: Alasdair Davies. (f) Weddell seal with a satellite relay data logger on its head with CTD (conductivity, temperature, and depth) sensor, part of the IMOS system. Photograph: Rob Harcourt. (g) Traditional manual VHF radio tracking of orange-bellied parrots (inset, with antenna visible of tail-mounted transmitter). Photographs: Zoos Victoria. (h) Bespoke radio-tracking UAV (Wildlife Drones) deployed for automated VHF tracking of orange-bellied parrots. Photograph: Zoos Victoria. (i) Birds can carry small monitoring equipment attached to a tiny harness—in this image, a light-based geolocator (GDL2, 0.6g) and multisensor logger (GDL3-PAM, 1.4g; ambient light, atmospheric pressure, temperature, acceleration) Photographs: Swiss Ornithological Institute. (j) Automated monitoring of feeder visitation by the critically endangered helmeted honeyeaters using RFID microchips Photograph: Zoos Victoria. (k) Raspberry Pi–based low-cost time-lapse cameras to monitor penguin colonies in Antarctica, with pictures reviewed by citizen scientists on the Zooniverse platform. Photograph: Alasdair Davies. (l) Automated detection of Marsh deer from color photographs taken from a fixed-wing drone using a deep-learning model. Photograph: Ismael Brack.

Along this pipeline, we distinguish the following types of technology. (1) Components (e.g., electronic or mechanical) are the building blocks and will only be mentioned when relevant (e.g., sensors). (2) Devices (machines): Components and parts are assembled together to form devices, from the simple (e.g., camera trap) to the complex (e.g., satellite); this is typically, the lowest (most physical) level that is relevant for conservation. (3) Systems: Several devices must often work together to achieve a given functionality (e.g., a wireless sensor network includes several types of devices). (4) Computers and processing: the backbone of most conservation technology, including storing, retrieving, manipulating, processing and visualizing data and derived information. (5) Software and data management: Software (e.g., computer programs and apps) provides the brains that allow devices to fulfill their potential. Most modern electronic devices require some control software, either directly interacting with the user (e.g., in a laptop) or running independently (e.g., data processing software within a GPS unit). (6) Algorithms and methods: The highest level of abstraction, including signal processing and data analysis, these are typically implemented as software running on computing devices. (7) Services: Modern technology is inherently associated with some level of service provision. For example, cellular networks require dedicated large-scale infrastructure and complex computing and software systems; from the user's perspective, connectivity is a service that allows using devices such as mobile phones to communicate and access the Internet. Web and cloud services (data storage, processing and analysis of large data sets) are becoming increasingly popular. Figure 2 illustrates a selection of technologies deployed for conservation purposes.

**Figure 3.**
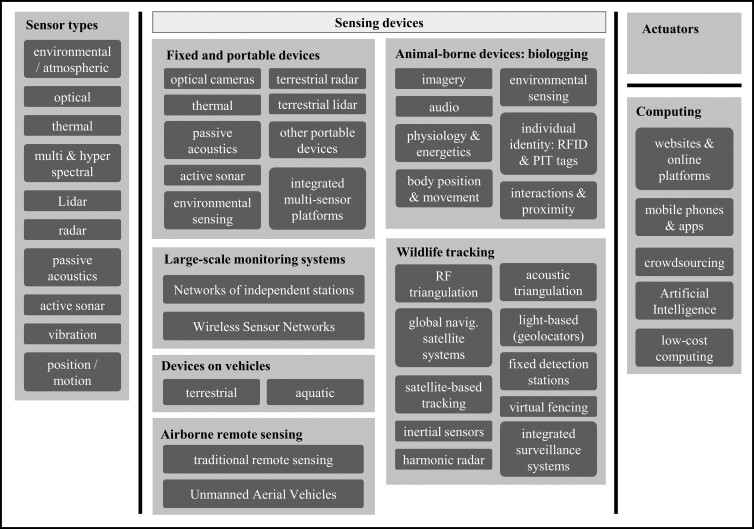
Index of technologies covered in this article, listed in the same order as in the article. The light and dark gray boxes correspond to sections and subsections.

We start with an overview of different relevant sensors types, often essential elements for many technologies. We then describe technologies that deploy sensors in devices that are fixed or portable, including large-scale networks; attached to vehicles (terrestrial or aquatic) or airborne, including satellites; attached to animals (biologging); or used to track moving wildlife. The last sections are dedicated to actuators and computing. Figure 3 shows a visual index of all the technologies covered by this article. We provide examples of conservation applications, both classic and novel whenever relevant, as well as a brief introduction to the principles behind a technology when deemed relevant for comprehension.

## Sensor types

One of the main purposes of using technology is to detect or gather data on wildlife, habitats, humans or their activities. This requires sensing abiotic, biotic and anthropic components of the environment. Most sensors are electronic components that convert a physical or chemical magnitude into an electric signal (either a current or voltage) whose value (or variation over time) can be measured, displayed, stored or further manipulated. We often use the term sensor to refer to a complete device (e.g., a camera trap as an optical sensor rather than the actual CMOS electronic component within it).

### Environmental and atmospheric sensors

Sensors exist to measure different physical and chemical magnitudes that reflect relevant aspects of environments (e.g., soils, aquatic) and the atmosphere, from temperature to volatile compounds (for some examples, see table 1; see a list of sensors in agriculture in Aqeel-ur-Rehman et al. 2014). Often, several sensors are integrated into a single electronic component (e.g., temperature and humidity sensor) or device (e.g., pipe::scan water quality sensor; www.s-can.at/news/item/176-the-new-pipescan). Most of these sensor types have been developed for industrial or agricultural applications, or atmospheric studies.

**Table 1.** Examples of environmental and atmospheric sensors.

Magnitude sensed or measured	Example of sensor
Temperature	Thermistors (resistance that varies with temperature)
Moisture content in soil or air (relative humidity), dew point	Electronic hygrometers (changes in capacitance or resistance)
Magnetic field (and its variation)	Magnetometers; the most common type are small solid-state Hall effect sensors (easily integrated in consumer electronics to sense the position of accessories)
Gas concentration (e.g., nitrogen dioxide, carbon monoxide, carbon dioxide, alcohol)	Gas detectors (e.g., based on electrochemical measurements or infrared)
Air quality, including particulate matter and volatile organic compounds, to monitor airborne pollutants	Air quality sensors (aka air pollution sensors)
Water quality, including physical properties (conductance, turbidity, color) and concentration of substances (e.g., salinity, pH, dissolved organic carbon, dissolved oxygen)	Water quality probes (often measure several magnitudes)
Volatile compounds	Electronic noses can recognize specific volatile compounds, or combinations of them (using sensor arrays). They require pattern-recognition algorithms trained to recognize target compounds. Some systems (e.g., Cyranose; www.sensigent.com/products/cyranose.html) have arrays of sensors that can be trained to detect different chemical profiles.

### Optical sensors

**Figure 4.**
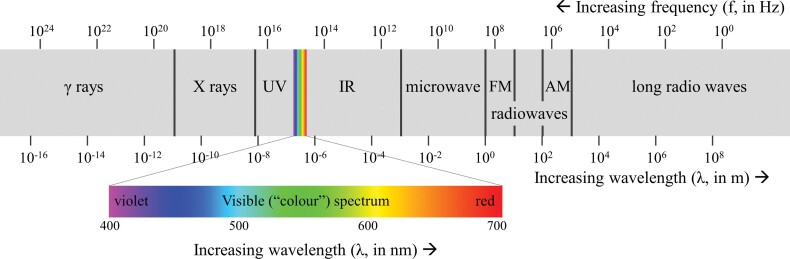
Electromagnetic spectrum, with visible light expanded. Wavelength (in measures of distance) is the inverse of frequency (in hertz). Source: Adapted from Philip Ronan (license CC BY-SA 3.0).

Optical sensors react to light, usually referring to the visual (color) part of the spectrum (wavelengths of approximately 400–700 nanometers [nm]; figure 4) but often encompassing ultraviolet (approximately 10–400 nm) and infrared (approximately 700 nm–1000 micrometers [μm]) wavelengths.

Optical sensors range from simple electronic components that react to incident light (e.g., a light-dependent resistor) to more complex optical systems (e.g., digital camera CCD image sensor). Sensors measuring light intensity (radiance) in different frequencies are often called *spectrometers* (sometimes *radiometers*, when outside the visible spectrum). The most common application is in interpreting the images generated, but other applications exist (e.g., derive geographic latitude; see the “Wildlife tracking” section). Spectrometry can be used to study the chemical composition of a substance.

Dedicated ultraviolet (UV) sensors exist, but even normal digital camera sensors react to UV light. Although they are uncommon, there are imagers than can directly apply UV for conservation purposes (e.g., to detect white-nose syndrome in bats; Turner et al. 2014). The infrared (IR) band is broad and includes electromagnetic radiation of very different properties. Beyond imaging, IR light can be used to measure distances (e.g., rangefinders used in distance sampling surveys to estimate population density; Thomas et al. 2010) and in 3D scanning (e.g., mangrove roots; Kamal et al. 2014). The *near infrared* band (NIR; 700–1100 nm) typically conveys information uncorrelated with visible-spectrum images. NIR's characteristics are often important in remote sensing applications: Clear sky and water absorb NIR, whereas healthy vegetation reflects it, helping delineating habitats and assessing vegetation health. Some digital cameras can be modified to capture NIR. NIR is also used in night vision imagers (which are different from thermal imaging), which provide images even in low-light conditions, often assisted by emitted NIR light (e.g., “night mode” in some video cameras).

### Thermal sensors

Thermal radiation (heat) is emitted by an object by the vibration of its molecules at a given temperature. A section of the infrared spectrum called thermal infrared (TIR) is particularly useful for monitoring wildlife, because animals and plants radiate in this band (and not in visible light) at their normal temperature; they are therefore detectable at night or in limited visibility conditions. Thermal imagers (or thermographic cameras) transform TIR radiation (3–14 μm) into an electrical signal that can form an image in a display or in a digital file. The most commonly used thermal sensors in consumer-grade devices are uncooled silicon-based microbolometers. TIR imagers and sensors are of much lower resolution than visible-light digital sensors (0.3 megapixels for a typical high-end commercial thermal sensor versus 80 megapixels for a high-end digital camera). Thermal sensors (including cameras, scopes, goggles, rifle scopes; handheld and airborne) have extensive industrial use (construction, electronics, firefighting). Low-cost, low-resolution (i.e., 160 ×  120 pixels) imagers are now available as mobile phones accessories (e.g., FLIR ONE range; www.flir.com/flirone) or for DIY electronics (e.g., FLIR Lepton Dev Kit; www.sparkfun.com/products/14654), opening the door for open-source options. Havens and Sharp (2016) provide a technical and practical introduction to thermal imagers (and night vision cameras).

### Multispectral and hyperspectral sensors

Some spectrometers measure the energy in many different bands across a broad part of the electromagnetic spectrum. They are often deployed for remote sensing purposes onboard planes or satellites. Multispectral sensors (e.g., the Operational Land Imager onboard the Landsat 8 satellite) measure several (approximately 3–15) discrete spectral bands. Hyperspectral sensors measure many more (e.g., hundreds) of narrower contiguous spectral bands, often across broad parts of the electromagnetic spectrum (e.g., NASA's Hyperion spectrometer onboard the EO-1 satellite: 220 contiguous 10 nm bands in 0.4–2.5 µm). The higher spectral resolution can allow the detection of landscape features; for example, multispectral images could help locate forest areas, whereas hyperspectral images could map specific tree species (Miyoshi et al. 2020).

### Lidar

Lidar (short for *light detection and ranging*, also called *laser altimetry*) is an active surveying method in which a laser is emitted toward an object (e.g., the ground, from an instrument onboard a plane) and its reflection measured by the same instrument. The time between emission and reception can be processed to obtain high-resolution information on the three-dimensional structure of the target. Different wavelengths (UV, visible, IR) can achieve different spatial resolutions (down to very small scales, such as centimeter scale), depending on the instrument and deployment. Airborne lidar can cover large spatial extents, but terrestrial (fixed) applications exist (e.g., to characterize forest structure). Bathymetric lidar to map underwater terrain uses different wavelengths to penetrate water. Melin and colleagues (2017) offer a good introduction to lidar focused on conservation applications.

### Radar

The electromagnetic spectrum beyond TIR is typically referred to as radio waves (figure 4). Beyond radio communications, radio waves are the basis for radar (for *radio detection and ranging*), an active sensing method for remote sensing. The two basic types are pulse Doppler radar (based on the frequency shift induced by moving objects to detect location and speed) and pulse echo radar (based on the time of flight to derive distance). Stationary radar systems are used to detect the location and speed of objects (e.g., maritime, aviation, or weather radars); they can also detect animals (e.g., birds; Gauthreaux and Belser 2003) or can be used for imaging purposes (deployed on a plane or satellite, for studying landscapes or deriving topography—e.g., digital elevation models). Portable low-power radars exist (www.flir.com.au/surveillance/display/?id = 64731), including boards for DIY development (e.g., detection up to 10 meters [m]; BumbleBee, samraksh.com/index.php/products/all-products/32-product-pages/products-sensors/71-bumblebee-radar).

### Passive acoustic sensors

Sound is a vibration that propagates through a medium (air, water, or a solid) as a pressure wave. Animal species are sensitive to different sound frequencies (hearing range), from very low (infrasound, below 20 Hz; e.g., elephants) to very high (ultrasound, above 20 kHz; e.g., bats). Sound can be sensed using microphones or hydrophones (underwater). Different sensing technologies are used depending on the desired characteristics (sound quality, sensitivity, directionality, size, cost). Sound recording devices have evolved from the traditional tape recorders to small (e.g., AudioMoth, www.openacousticdevices.info; Song Meter Micro, www.wildlifeacoustics.com/products/song-meter-micro) sound loggers. Passive acoustic sensors can also be used to locate wildlife (see the “Acoustic triangulation” section, under “Wildlife tracking”). Passive acoustic monitoring can also occur underwater; it is often called *passive sonar* when the objective is to detect acoustic signals.

### Active sonar

Sonar (for *sound navigation and ranging*) is a specific application of acoustics to communicate, navigate, detect or measure target objects, particularly underwater. Unlike in passive acoustics, active sonar systems listen to the reflection of an emitted sound pulse (ping) on a target object. A variety of technologies exist with different characteristics (e.g., spatial resolution, distance) and frequencies (from infra to ultrasounds). These include multibeam echosounders (www.kongsberg.com/maritime/products/mapping-systems/mapping-systems/multibeam-echo-sounders), higher-resolution, short-range “acoustic cameras” (e.g., DIDSON; www.soundmetrics.com/Products/DIDSON-Sonars) and ocean acoustic waveguide remote sensing (for instantaneous imaging and continuous monitoring of fish populations over continental-shelf-scale areas; Jagannathan et al. 2009). Beyond classic military and civilian maritime applications, sonar is used to detect fish schools and can estimate biomass in fisheries studies, map depth (bathymetry), and help navigation by automated vehicles (e.g., robots).

### Vibration sensors

Geophones convert ground movement (vibration, vertical or horizontal) into an electric signal, using different technologies. Their principal use is in seismology (at very large scales), but they can also be used to detect vibration created by smaller objects at more local scales, such as the vibrations emitted by elephants (Mortimer et al. 2018).

### Position and motion sensors

Some sensors can measure locally a variety of magnitudes related to movement and position. An inexhaustive list includes accelerometers (acceleration), gyroscopes (rotation and tilt), rate gyroscopes (angular velocity), magnetometers (magnetic field, heading; can be used as compass). They have many applications, including robotics, gaming (gesture detection), image stabilization in cameras. Inertial measurement units (IMU) or inertial navigation systems (INS) integrate several position and motion sensors, facilitating stable flight (e.g., drone) and navigation by dead reckoning (continuous update of the position, orientation and velocity). Motion can also be detected on the basis of changes in the amount of heat received by a sensor (e.g., passive infrared [PIR] motion sensors in some security cameras and wildlife camera traps) or algorithmically by analysis of images (see the “Computing” section).

## Fixed and portable devices

This section covers a range of devices that are either portable or can be deployed in fixed locations.

### Optical cameras

Camera traps (or remotely triggered cameras) can capture still pictures or videos after being triggered by an animal. They have become essential tools for monitoring many (particularly terrestrial) species. This is a mature technology with a long history of development (more than100 years) and a rich literature, including dedicated books (e.g., O'Connell et al. 2011), reviews of devices, applications and associated statistical methods (e.g., Hamel et al. 2013, Burton et al. 2015), and a recent comprehensive review providing best practices (Wearn and Glover-Kapfer 2017).

Modern camera traps typically consist of a digital image sensor, a PIR sensor (to detect animals in front of the camera), and auxiliary electronics (e.g., memory card) and protective casing. Price ($50–$1000) depends on many aspects (e.g., image quality, triggering delay, and capabilities). Camera traps have been used in conservation for different purposes, including (table 7-2 in Wearn and Glover-Kapfer 2017) monitoring wildlife population status (e.g., recording presence or absence, detection rate, or specific individuals with artificial or natural markings; searching for rare species; estimating biodiversity, including rapid assessments; studying habitat preferences and behavior; detecting poachers). They are often used for terrestrial animals of mid-size to large (e.g., cat to deer size) but can also detect smaller animals (e.g., rodents) at closer range. Although challenging, some studies have used them for arboreal animals. Ectotherms are also challenging to detect using a PIR sensor (body often close to ambient temperature).

More advanced camera trap features include infrared flash (night illumination without disturbing animals), wireless connectivity (pictures sent through cellular network or Wi-Fi), networked cameras (e.g., BuckEye Cam; www.buckeyecameras.com), and remote checks of camera status. New intelligent camera traps (in-device image processing; see the “Computing” section) have important conservation applications (e.g., detecting humans for antipoaching, PoacherCam; www.panthera.org/conservation-technology/poachercam) but are not yet widespread. A few open-source camera-trap systems have been developed, using off-the-shelf components (Williams et al. 2014), sometimes geared toward education (e.g., Naturebytes; naturebytes.org). The future directions of camera-trapping technology were recently reviewed (Glover‐Kapfer et al. 2019).

Cameras are also used underwater. Baited remote underwater video stations (BRUVS) are becoming a popular tool to study demersal and nektonic communities, particularly fish (Langlois et al. 2020 provided a field and annotation guide). They most often work in a non-triggered way, with continuous or programmed recordings. Underwater triggered camera trapping is less common (Williams et al. 2014), with challenges including low light levels (particularly less than 40m), high pressure, and image triggering relying on image processing algorithms (infrared triggering does not work).

Managing, visualizing, and sorting massive amounts of images can become a resource-consuming task, even the limiting factor for a project. Software programs, applications, and algorithms (see the “Computing” section) have been developed recently to alleviate this burden, including automated sorting (e.g., images with no animals), automated species identification, online sharing to crowdsource human-based species identification.

### Thermal imaging

The contrast between the heat emitted by animals and their immediate surroundings can help detect them efficiently and unobtrusively, particularly under some conditions (e.g., at night, with cryptic background or hidden by vegetation). TIR imaging has long been used to detect wildlife and monitor animal populations (Havens and Sharp 2016), particularly for large-bodied endotherms (e.g., ungulates); this is linked to hunting, one of the main uses of thermal imaging (handheld devices and rifle scopes). It has also been used for small mammals (e.g., rodents; Boonstra et al. 1994), birds (McCafferty 2013), nests, and cave roosts (Hristov et al. 2008). Infrared radiation attenuates very quickly in water, so thermal monitoring has been rather limited in aquatic environments (e.g., surfacing marine mammals and intertidal invertebrates; Lathlean and Seuront 2014). Some studies have shown thermal detection to be more efficient than spotlighting (Focardi et al. 2001), but this will depend on each case (species, habitat, environmental conditions). Thermal sensing is not always intuitive; many aspects influence the relationship between body temperature and how much TIR radiation hits a thermal sensor (e.g., ambient temperature, insulation by fur, surface temperature versus core body temperature, distance to target, field of view of the lens); pilot studies can help test whether the approach is sensible for a specific purpose.

Thermal imaging has been used in a variety of contexts beyond wildlife monitoring, including research on migrations (McCafferty 2013), behavior (e.g., flight patterns; Hristov et al. 2008), welfare and disease diagnosis (Cilulko et al. 2013), to avoid killing of animals (e.g., farmland bird nests, fawns) during mowing (Steen et al. 2012), to detect wind farm collisions of birds (Desholm et al. 2006).

The growing literature on the use of TIR for wildlife studies and monitoring includes a dedicated book (Havens and Sharp 2016). The use of this technology is likely to increase in the coming years driven by the decreasing cost of lightweight handheld devices.

### Passive acoustics

Monitoring wildlife on the basis of the sounds they produce has a long tradition in ecology and conservation. Initially relying on human hearing and tape recorders, the discipline flourished with the development of modern passive acoustic monitoring (PAM) on the basis of acoustic loggers, electronic devices left unattended in the field for extended periods, recording sound at preprogrammed intervals or in response to acoustic triggers. Acoustic monitoring is a booming discipline and modern low-cost automated PAM could enable biodiversity monitoring at unprecedented spatial and temporal scales. Acoustic monitoring has a rich literature on applications (different species, locations) and technologies (e.g., automated species identification, statistical methods). A recent overview (Browning et al. 2017) covers from technology to survey design and data analysis; Sugai and colleagues (2019) offered a recent review and perspective.

Many animal taxa contain vocal species, notably many birds, frogs, insects, mammals (e.g., primates, rodents, cetaceans, and bats use ultrasonic vocalizations, and elephants use infrasonic) and even fish. Some illegal activities (e.g., chainsaws, gunshots) and invasive species (e.g., cane toads, Hu et al. 2009) can be detected acoustically. Sound propagates well underwater and PAM is used to monitor cetaceans and, increasingly, fish and invertebrates. PAM, particularly with recent cheaper devices, can be used to search for rare, elusive or sparse species (or their threats) in very large landscapes (Hill et al. 2018). Trends in species occupancy and abundance, and biodiversity indices (e.g., species richness) can be estimated from PAM data, often relying on robust statistical methods (e.g., spatially explicit capture–recapture, Royle et al. 2013; occupancy detection, Campos-Cerqueira and Aide 2016) to deal with imperfect detection (including false positives, particularly with automated identification). The recent discipline of soundscape ecology (Pijanowski et al. 2011) studies the relationship between ecological processes and the soundscape (spatiotemporal variation of sounds in a landscape), reflecting important ecosystem processes and human activities. Soundscape studies do not require identification of individual species but still generate large amounts of data. Methods have been developed to summarize soundscape properties (acoustic indices, Sueur et al. 2014) and facilitate interpretation (Towsey et al. 2014). Soundscape monitoring may provide affordable large-scale surveillance of ecosystem health (Farina 2014) and surrogates for monitoring biodiversity (Burivalova et al. 2018).

PAM traditionally relied on expensive commercial equipment ($800–$1200 per unit), labor-intensive retrievals of memory cards and manual checking of recordings for target sounds or training recognizer algorithms. The discipline is now reaching a new level of maturity, with cheaper and more intelligent equipment. Low-cost open-source devices have been proposed (e.g., DIY, Whytock and Christie 2016; integrated, Wijers et al. 2019). The promising AudioMoth (Hill et al. 2018), with its key characteristics (open-source design, bespoke programming, low energy consumption, a low-cost of approximately $80), represents a milestone in PAM technology. Automated acoustic species recognition has vastly improved, reducing labor and enabling real-time species detection (Aide et al. 2013). Modern electronics could allow in-device detection of species or events in real time, critical for some conservation applications (e.g., poacher gunshots; Wrege et al. 2017). Automating the data pipeline (handling, storing, processing) is essential to operationalize PAM at scale, given the massive amounts of data PAM typically generates, and some end-to-end solutions (e.g., ARBIMON includes acoustic stations wirelessly connected to a data repository; Aide et al. 2013) and backend support (e.g., Ecosounds online repository, www.ecosounds.org; Wimmer et al. 2013) have been proposed.

### Active sonar

Active sonar has a long-standing tradition in seafloor mapping and fisheries, with some applications to conservation including understanding the impact of fishing practices (Lucchetti and Sala 2012), surveying for threatened species (Flowers and Hightower 2013) and studying marine fauna (Giorli and Au 2017). These devices are often towed from a boat or deployed in buoys. Acoustic cameras (e.g., DIDSON and its successor ARIS) achieve sound-based real-time near-video imaging at short distances of tens of meters (Moursund et al. 2003). They are portable devices (including a handheld version for divers with a mask-mounted display; soundmetrics.com/Products/­­ARIS-Sonars/ARIS-Defender-3000) and can substitute optical cameras for detecting, identifying and counting fish species in turbid waters.

### Environmental sensing

In the context of conservation, the most common use of many environmental and atmospheric sensors is to monitor environmental quality and detect changes in environmental conditions, mostly as background surveillance monitoring. For example, long-term ecosystem monitoring sites of NEON (the US National Ecological Observatory Network) deploy several automated environmental sensors (Thorpe et al. 2016), including water quality (e.g., dissolved oxygen, pH, nitrate), atmospheric (e.g., solar radiation, wind speed) or in the soil (e.g., temperature and moisture). iButton data loggers (www.maximintegrated.com/en/products/ibutton/data-loggers.html) are an increasingly popular small, low-cost, robust sensor that can log temperature or humidity in the field for long periods of time. Buoys and bottom-tethered devices are used to deploy environmental sensors at sea, often as multisensor platforms (e.g., the United Kingdom's autonomous SmartBuoys; www.cefas.co.uk/data-and-publications/smartbuoys), providing long-term data to assess eutrophication, environmental variability and ground-truth satellite images. FieldKit (www.fieldkit.org), an open-source modular environmental sensing platform has just been released.

Vibration created by movement and low-frequency sounds can be recorded using geophones and processed using seismic signal processing techniques. It has been used to detect large mammals such as elephants (Mortimer et al. 2018), with potential for monitoring some species (Wood et al. 2005). Geophones have been used to study the behavior of small fossorial animals (Narins et al. 1997) and could potentially be used for monitoring. Early detection of large species that produce ground vibrations (e.g., elephants) can be used to reduce human–wildlife conflict (Anastácio et al. 2018). Some human activities could be tracked with geophones (e.g., mining blasts (Wrege et al. 2010) or vehicles associated to poaching or logging).

Subterranean activity can also be tracked using magneto-inductive localization by measuring magnetic field strength generated by antennas (e.g., badger tracking over 15 ×  20-m area; Noonan et al. 2015).

### Terrestrial radar

Ground-based stationary radars have been used to detect or track flying animals. The basic idea is not new (Vaughn 1985) and early radar ornithology provided the first sound evidence of nocturnal bird migration. The discipline has seen a revival, particularly following the expansion of wind turbines (to model collision risk with flying birds; Desholm et al. 2006), as well as access to cheaper equipment and online data from weather radar, and modern computing for dealing with large data volumes. Low-powered surveillance radars, which can detect bird movements within a few kilometers, have been used to study migrations for several decades. Long-range, powerful surveillance radars (including airport, weather station and military radars), which can detect birds at much larger ranges (100–240 kilometers [km]), have been used to study migration (Gauthreaux and Belser 2003) and estimate migration numbers (Dokter et al. 2018, with weather radars) at continental scales.

With Doppler weather data (good coverage in the United States and Europe) now freely accessible online, international research collaboration is growing in this area (e.g., ENRAM; www.enram.eu). Small purpose-built vertical-looking radar (e.g., BirdScan; swiss-birdradar.com) can detect individual birds, bats, and insects flying over it and can be used for research on migrations (e.g., average flight direction and speed), including at high altitude and at night (Chapman et al. 2003), environmental consulting studies (e.g., planning infrastructure projects such as wind turbines), and conservation (e.g., temporary shutdown of wind turbines when large flocks detected). The taxon can sometimes be derived from radar signal analysis (Zaugg et al. 2008). A recent study compares the strengths and weaknesses of different radar types (from Doppler weather radar to dedicated bird radars) operated at the same location (Nilsson et al. 2018). Some conservation-focused applications exist (Gauthreaux and Belser 2003), including understanding bird migration patterns and stopover areas at continental scales. This technology is likely to see most conservation application for flying species, including insects (Drake and Reynolds 2012).

### Terrestrial lidar

Portable terrestrial lidar (or terrestrial laser scanning, TLS) devices allow rapid collection of high-resolution (less than 1 centimeter [cm]) 3D spatial structure of natural habitats (Vierling et al. 2008). Portable devices include, for example, Echidna lidar (approximately 20 kilograms [kg]) as well as lighter options, optimized for rapid scanning and portability, such as the Compact Biomass Lidar (3.4 kg) or the handheld Zebedee (see links in Paynter et al. 2016). TLS data can help ground-truth areal lidar data, or conversely aerial data can scale up the more detailed parameters measured with ground-based lidar. Estimating tree structure can help monitor ecosystem condition and measure tropical forest carbon stocks (Tanago et al. 2018).

### Other portable devices

Smartphones are currently widely available and usually carry several sensors beyond the obvious camera and microphone, including proximity (infrared or magnetic Hall effect), ambient light, atmospheric pressure, magnetometer (magnetic compass), accelerometer, gyroscope, temperature, and GPS. Moreover, they can send data remotely thanks to in-built connectivity (cellular and wifi). Some of these sensors are only available in higher-end phone models, but even a microphone paired with wireless connectivity can be an effective conservation tool (e.g., Rainforest Connection developed automated acoustic detection of illegal activities—machinery—on the basis of discarded phones and sound analysis in a cloud server; rfcx.org/our_work). Mobile phones as connected multisensor platforms may allow new ideas with great potential for conservation, such as *crowdsourcing* (see the “Computing” section).

Electronic noses have detected wildlife disease from carefully prepared lab samples (e.g., tuberculosis in badgers; Fend et al. 2005); applicability to field situations are currently being tested (Doty et al. 2020).

### Integrated multisensor platforms

Sometimes several sensors are combined in the same device or station. This is typical for example of environmental sensing and can even scale up to very large networks (see the “Large-scale monitoring systems” section). There is an increasing interest in integrating wildlife monitoring sensors that would have typically been deployed independently. For example, the Instant Detect (www.zsl.org/conservation/conservation-initiatives/conservation-technology/instant-detect) platform expands the traditional camera trap to accommodate other sensors (e.g., acoustic), with real-time data communication via satellite. It has been trialed to detect small illegal fishing vessels (www.zsl.org/conservation/conservation-initiatives/conservation-technology/detecting-illegal-fishing-vessels). The AmiBio project (www.evolving-science.com/research-grants/amibio-automatic-acoustic-monitoring-and-inventorying-biodiversity-00485) integrates acoustics with weather data. Automated biodiversity sampling stations are being designed (e.g., project AMMOD, www.zfmk.de/en/research/projects/ammod-a-weatherstation-counting-species-diversity, which includes DNA identification of insects, pollen and airborne spores; image recognition of birds, mammals, and nocturnal insects; acoustic detection of birds, bats, and grasshoppers; and analysis of biogenic scents). Other multisensor platforms include intensively monitored nest boxes (Zárybnická et al. 2016).

## Large-scale monitoring systems

The last couple of decades have seen an increasing use of networks of advanced sensors in ecological studies (Porter et al. 2009), and the integration of several technologies forming complete monitoring or surveillance systems is possibly one of the most promising areas of conservation technology (Marvin et al. 2016), allowing greater spatial and temporal resolution. *System* in the present article refers to several devices interconnected into a single functional entity, most often with some degree of control software.

### Networks of independent stations

A simple system may consist of a network of individual stations (see the multisensor examples in the previous section) that may be coordinated but do not directly communicate with each other wirelessly. Their strength resides in the mass of data gathered over time at many different locations, which can be jointly analyzed. Some of the largest such networks run at the continental (e.g., NEON in the United States; www.neonscience.org) or planetary scale (e.g., ILTER; www.ilter.network), with a strong emphasis on research collaboration and monitoring infrastructure.

### Wireless sensor networks

Wireless sensor networks (WSN) can relay data back to a central facility wirelessly (e.g., satellite or cellular networks), and sometimes even let the sensor nodes talk to each other. The review by Porter and colleagues (2005) identified five reasons for using WSN: high observation frequency, wider area coverage, unobtrusive observation, real-time data acquisition, bidirectional communication, allowing control of sensor functions. Sensors can be deployed following different spatial configurations, depending on the hierarchy of nodes: Some are simple sensors, others can route data and commands toward a central control system. Some WSN nodes can be carried by animals (see the “Biologging” section). Sensor-to-sensor connectivity allows new functionalities (Collins et al. 2006), including *mesh networks* (nodes can relay data dynamically, making the system robust to failures in a single sensor), self-diagnosis (e.g., sensor failure detected by nearby sensors), in-device processing (e.g., remove outlier data by comparing with nearby sensors), automated adaptive sampling to react in real time to locally sensed events. Data relaying is the most common functionality in current WSNs; others are still under exploration. Optimal WSN configuration and control is an active area of research in engineering and computer science.

WSNs are a promising technology for environmental monitoring, with plenty of potential for ecological research (Porter et al. 2005, 2009, Collins et al. 2006), including in aquatic environments (for a review, see Xu et al. 2014). Although often the sensors are environmental or climatic, they can also include cameras or acoustic sensors (e.g., Cai et al. 2007). Conservation-specific WSNs are still rare, but examples exist, some of them experimental. These include biosecurity (for a review, see Jurdak et al. 2015), including invasive species detection (e.g., cane toads; Hu et al. 2009) and for monitoring remote locations (e.g., seabirds colonies; McKown et al. 2012).

## Devices on vehicles: Terrestrial and aquatic

The typical aim of having a sensor on a terrestrial or aquatic vehicle is to cover more ground than a walking human could, by relying on greater speed or autonomy. In contrast, airborne remote sensing has the added benefit of the sensor itself covering a greater area, thanks to a greater distance between sensor and sensed area.

### Terrestrial vehicles

Some handheld sensors have been used for wildlife monitoring from cars (either fixed to the vehicle or held by a passenger). This is typically done to cover longer distances while maintaining a good chance to detect wildlife (e.g., handheld thermal imager from a slow car; Morelle et al. 2012). Cars increase coverage but not autonomy (a human driver is still needed). We consider autonomous terrestrial vehicles (cars and robots) to be an emerging technology with no current conservation applications; they must overcome the challenges of moving through natural environments.

### Aquatic vehicles

Cameras and sensors deployed from vessels or attached to manned submersibles have long been used to study underwater environmental conditions and biodiversity, including for conservation purposes (e.g., imaging for marine conservation planning; Schlacher et al. 2010). Missions are expensive and limited in area coverage; we concentrate in the present article on discussing a relatively recent technology: unmanned aquatic vehicles, which add autonomy and can reach challenging locations. Beyond the generic term *drone*, the somehow overlapping terminology includes *remotely operated vehicles* (tethered and remotely controlled from the surface; Shepherd 2001), *autonomous underwater vehicles* (AUVs, which may have some autonomous behavior), *unmanned underwater vehicles*, *autonomous surface vessels* (www.aims.gov.au/advanced-observation-technologies/autonomous-surface-vessels), *unmanned surface vehicles* (*sailing drones* for long deployments; www.saildrone.com), and *underwater gliders* (which use small changes in buoyancy to move up and down; dx.doi.org/10.1051/matecconf/20141302020). The main differences include whether the vehicle operates above or below the surface, whether it is remotely controlled or autonomous, its type of movement (gliding, floating, rolling on the sea floor), and its propulsion system (wind, solar, fuel, or tethered for electricity supply over short distances). One or more sensors may be integrated within the body or on their surface, or attached to an extension arm. Many different sensors have been deployed, from cameras to sonar and environmental and chemical sensors (e.g., multisensor oceanic drone Saildrone; www.saildrone.com), and include suction devices to capture specimens. Underwater drones have traditionally been large expensive experimental (or military) devices relying on important infrastructure for deployment. The recent emergence of smaller, autonomous, cheaper (even DIY; www.seaperch.org/index) options might allow a resurgence in their use.

Aquatic drones can be deployed for a variety of tasks, depending on their autonomy and characteristics. A few examples related to conservation and biodiversity include biodiversity exploration (e.g., new species; Raskoff and Matsumoto 2004) and monitoring (e.g., video and sonar coral reef surveys; Singh et al. 2004), document threats (e.g., trawl fishing damage to deep-sea coral reefs; Hall-Spencer et al. 2002), invasive species management (e.g., automated detection of invasive starfish; www.araa.asn.au/acra/acra2005/papers/clement.pdf), reducing the impact on marine mammals (e.g., hydrophone-based automated localization; www.navaldrones.com/ZRay.html).

## Airborne remote sensing

This section describes sensing devices that are used from airborne vehicles. The first part covers traditional remote sensing, from manned vehicles (like planes or helicopters) to satellites (including nanosatellites, a newer option), while we dedicate the second part to unmanned aerial vehicles (UAVs), a remote sensing technology that is rapidly becoming popular.

### Traditional remote sensing

Although *remote sensing* literally refers to the study of objects without physical contact, the term is normally reserved for when observations are taken from a long distance, including monitoring from airborne or spaceborne sensors. Manned planes and helicopters have long tradition in wildlife monitoring, by humans or onboard sensors (e.g., distance sampling transects for cetaceans). Large scale (even global) ground coverage is obtained by sensors on board satellites; these programs are extremely expensive and traditionally handled by governmental organizations (e.g., NASA) or large private companies (but see the “Nanosatellites” section below). Images are then distributed as a product, often after substantial image processing. After acquiring images, advanced image processing skills are still often required for postprocessing and analysis. Satellite-based remote sensing is characterized by temporal (how often an area is covered), spatial (how much area covered) and spectral (what frequency bands) resolutions. Satellite-based remote sensing is a huge field, with a long history in environmental applications. There is a well-developed literature around environmental remote sensing (Wang et al. 2010, Kuenzer et al. 2014), and ecological applications more specifically (reviewed in Pettorelli et al. 2014). In the present article, we give illustrative examples of biodiversity-related applications. Satellite-based remote sensing is probably one of the greatest technological leaps for studying biodiversity.

*Color or NIR images: *Traditionally, most biodiversity studies using satellite imagery relate remotely sensed habitat measures to species preferences (for a review, see Leyequien et al. 2007), including in marine environments (e.g., coral reefs, Xu and Zhao 2014). Different spectral bands quantify information that can be related to some aspect of biodiversity (e.g., geophysical variables such as sea-surface temperature), indices (e.g., normalized difference vegetation index), thematic variables (land or water cover), topographic variables (surface roughness), and image textures (patch size, habitat fragmentation, and connectivity). These can help map habitat boundaries, estimate habitat preferences and species distributions, assess vegetation and habitat status, locate human-induced pressures, and threats. Often the focus is on tracking their temporal variation (e.g., map deforestation). Detecting and counting animals is the most common application of aircraft-based monitoring; the coarser ­spatial resolution has traditionally limited the use of satellite imagery for this, although some examples exist, including through habitat modification by animals (e.g., bare ground around wombat warrens, Löffler and Margules 1980; fecal staining to count penguin colonies, Fretwell and Trathan 2009). Newer higher resolution sensors (e.g., 1.65 meters [m]; Geo-Eye satellite) allow direct individual counts but this is uncommon and relies on large size (e.g., large savannah mammals; Yang et al. 2014) or high contrast (e.g., ­albatrosses; Fretwell et al. 2017). High-resolution daily mapping of landscape based on large constellations of small satellites has great untapped potential for conservation (www.sciencemag.org/news/2017/02/flotilla-tiny-satellites-will-photograph-entire-earth-every-day).

*Multispectral or hyperspectral images: *Some well-known Earth-observing sensors are multispectral (e.g., satellite Landsat) or hyperspectral (Hyperion spectrometer in EO-1 satellite, NASA's aircraft-based AVIRIS). These provide more detail on vegetation, soils (geology, chemistry), and atmosphere than color or NIR sensors. Most applications relate directly to habitat, vegetation, and environmental conditions and only indirectly to assisting conservation. Hyperspectral imagery has been used for plant species identification, monitoring soil properties, mapping habitat, and assessing plant condition (Pettorelli et al. 2014). Plant communities can be mapped, even down to species level (Kuenzer et al. 2014), including invasives (Walsh et al. 2008).

*Lidar: *Airborne lidar has been used in ecology and conservation (for reviews, see Vierling et al. 2008 and Melin et al. 2017). It can characterize three-dimensional habitat structure in terrestrial and aquatic environments at high resolution over broad areas, with two benefits: replacing labor-intensive field measurements and measuring novel habitat characteristics. Habitat structure measurements (e.g., forest canopy, canopy cover, leaf area index) can generate predictors to model biodiversity, including species distributions (e.g., Goetz et al. 2007) and habitat quality. Airborne lidar can also assess land cover, topography, and hydrology. Mapping forest biomass can provide input into schemes for carbon emission reduction such as REDD+ (e.g., AToMS, several sensors; directory.eoportal.org/web/eoportal/airborne-sensors/atoms). Although not suited to monitor animals directly, lidar has been used indirectly (e.g., finding malleefowl mounds; www.nationalmalleefowl.com.au/uploads/pdfs/21_V%20Saffer_Use%20of%20LiDAR.pdf).

*Radar: *Data from airborne radar can also be used to derive proxies of vegetation height and structure, bringing complementary information. An important radar-derived product is the global high-resolution digital elevation model, produced in 2000 by the Shuttle Radar Topography Mission. It has become a standard in spatial distribution studies, with many important topography-related suitability predictors (slope, aspect, ruggedness) derived from it.

*Nanosatellites: *Satellites have traditionally been expensive to design, manufacture and launch into orbit. Increasingly smaller (and cheaper) satellites have been developed in the last decades, from miniature (100–500 kg) to micro- (10–100 kg) and nanosatellites (1–10 kg). CubeSats (nanosats) are of particular interest for conservation. They have a modular design (10 × 10 × 10-cm units) and are often built from commercial components (even open-source development kits; www.cubesatkit.com), and they offer more affordable access to space, and short project times of 9–24 months (Allan et al. 2018). CubeSats have started a revolution in Earth observation: large low-Earth orbit constellations allow covering a large part of the planet simultaneously, although at coarser resolution than the most advanced large satellites (Marvin et al. 2016). They promise near-real-time global monitoring at increasing resolution, allowing analysis of changes in ecosystems, land use, and threats (e.g., road construction, illegal fishing, oil spills), with great potential for conservation. For example, PlanetLabs (www.planet.com) images the entire Earth every day at 3–5 m resolution, with targeted monitoring at 72 cm (Boshuizen et al. 2014). Radar systems are also being deployed on nanosatellites experimentally (e.g., to detect and track on the ground objects irrespective of cloud cover; www.bbc.com/news/science-environment-43544211).

### Unmanned aerial vehicles

Unmanned aerial vehicles (UAVs) or remotely piloted aircraft systems (RPAS), often referred to as *drones*, are aircrafts without an onboard human pilot. UAVs can have different degrees of autonomy ranging from full human manual control to preplanned missions followed autonomously (GPS navigation), all the way to onboard autonomous decision-making allowing obstacle navigation (usually military or experimental systems). Research is underway to develop swarming drones (group flight with coordinated actions through drone-to-drone communication).

Most UAVs belong to two main types: fixed-wing (unmanned planes, propelled by an engine or, less commonly, as a glider) and multirotor (unmanned multicopters, with several pairs of engine-powered rotor blades). Other more experimental options exist (e.g., flapping ornithopters) but have not been used for conservation yet. These two types have well-known characteristics (Anderson and Gaston 2013) that often sit at the opposite end of trade-offs. Compared with multirotor, fixed-wing drones typically fly longer and are less noisy, but are unable to hover or fly at very slow speeds, require open spaces for takeoff and landing, and more training. From their military origins, the last 5–10 years have seen the emergence of a consumer market for affordable (approximately A$2000) to inexpensive (approximately A$200) drones, particularly ready-to-go quadcopters with very stable flight. Despite lower capabilities compared with military-grade ones, they still bring great potential for conservation applications. Open-source DIY options (e.g., ArduPilot of the DIYDrones community; diydrones.com/notes/ArduPilot) have been nurtured by a community of enthusiasts.

Drones offer some clear benefits: access to remote, dangerous or difficult locations, efficient large-area coverage, better or new vantage points (e.g., above), safer than manned flights. Compared with satellites, drones offer controlled revisit periods, low-altitude flights, and much lower operational costs (Anderson and Gaston 2013).

The use of drones in natural environments still faces important challenges (Hardin et al. 2019), including limited flight times of most consumer-level options (less than 30 minutes) and strong legal restrictions (though sometimes easier in natural areas). Field trials often find this technology difficult or unsuitable; only recently, evidence is starting to unravel their true potential to study and protect biodiversity, with field testing and comparing their efficiency with traditional survey methods (Linchant et al. 2015, Hodgson et al. 2018). UAVs won't be suitable for all species and locations, and some applications, particularly related to imagery (e.g., orthorectified images) require substantial expertise. Two other issues that require further consideration are the social and ethical issues of privacy (What is the impact of capturing video of humans living in the area? Sandbrook 2015) and animal welfare concerns (What is the impact of the drone on wildlife? Mulero-Pázmány et al. 2017).

Drones have been used in a variety of biodiversity-related applications, with a few general and review papers (Koh and Wich 2012, Anderson and Gaston 2013, Linchant et al. 2015) and a conservation-focused book (Wich and Koh 2018) offering insight. Some of these applications (reviewed in Christie et al. 2016) include monitoring marine and terrestrial wildlife, from orangutans to orcas, for population monitoring, detection of presence, or even individual identification and behavior studies; monitoring and mapping the status and changes of habitat, vegetation, and land use (e.g., to detect and measure illegal logging); managing threats on the ground (e.g., antipoaching surveillance). Organizations such as ConservationDrones (conservationdrones.org/mission) and the World Wildlife Fund (www.worldwildlife.org/projects/wildlife-crime-technology-project) have tested UAVs in a variety of applications. Reviews of nonconservation uses (e.g., environmental; Colomina and Molina 2014, Pajares 2015) can offer inspiration for novel conservation applications—for example, monitoring forest fires (Merino et al. 2015) and invasive species (e.g., ResQu drone; research.csiro.au/robotics/project-resqu).

Other sensors beyond color cameras are increasingly used, including thermal (Christiansen et al. 2014, Gonzalez et al. 2016), multispectral or hyperspectral, and even lidar or radar, although larger sensors require bigger UAVs. Advanced automated processing of images (see the “Computing” section) will greatly improve the usefulness of drone-based imagery (Dell et al. 2016). Drones could also help acoustic monitoring, with suspended microphones (Wilson et al. 2017) or deploying acoustic loggers in difficult places (e.g., forest canopy). Drones have been proposed as a data communication node (e.g., data retrieval while flying over a camera trap; Glover‐Kapfer et al. 2019). Many advanced applications are at research or early trial stages, including grasping objects in flight (Thomas et al. 2014) or perching (Doyle et al. 2013).

## Animal-borne devices: biologging and biotelemetry

*Biologging* refers to the collection of data from sensors located on or inside an animal. *Telemetry* refers to the automated transmission of remotely gathered data. Biologging is often coupled with telemetry technology, sometimes termed *biotelemetry* (Cooke et al. 2004a). *Telemetry* may also refer to the determination of an animal's position using data transmitted from the animal (e.g., a VHF—very high frequency—collar); such position telemetry is commonly called *wildlife tracking, radio tracking*, or *wildlife telemetry*. Biologging and tracking can lead to insights into animal health, ecology and behavior that may be critical for wildlife management and conservation. Overall, wildlife telemetry can be considered one of the areas of technology that has had a massive impact in conservation and ecology (Kays et al. 2015). Biotelemetry and tracking tags can be attached to wildlife externally (depending on the species, using collars and harnesses or glued to skin, scutes, fur, or feathers) or may be surgically implanted. To avoid the need for recapture, some tags can be programmed or remotely triggered for release, others can transmit data wirelessly to a receiver. Nevertheless, the capture, handling and carrying of tags may cause stress and changes in animal behavior, leading to ethical concerns about potential impacts of their (increasingly widespread) use (Cooke et al. 2017). Tags have been used with a variety of taxa, from large mammals to invertebrates, and sometimes provide insight extremely difficult to obtain through other observational approaches (e.g., diving sea mammals). Although used for decades, progress has been phenomenal in the last 10–15 years, including improved tools for data management, visualization, integration and analysis (Rutz and Hays 2009). Some telemetry tags can be expensive (well above $1000), which limits the number of individuals that can be tagged. Recently proposed open-source alternatives (e.g., Arribada's Horizon platform; blog.arribada.org/2019/12/08/new-horizons-open-access-argos-telemetry) could significantly reduce their price. Many limitations are still technological, including battery life, miniaturization, data transmission rates and sensor capabilities (Bograd et al. 2010).

This section deals with biologging and biotelemetry (i.e., data about the animal's body and environment), showcasing different types of animal-borne sensors; we treat location data separately in the next section (“Wildlife tracking”). Note these areas often overlap (some tags allow simultaneous tracking and biologging) and often better insight is obtained by merging biologging and position data.

### Overview of biologging and biotelemetry

Biologging allows gathering data on physiology, behavior, energetics, basic ecology, and interactions of free-ranging, undisturbed animals, and even the environments in which they live. This is achieved by sensing and recording a staggering variety of physical and chemical magnitudes, from temperature to blood flow, from image to sound, from body position to proximity to other individuals (see table 2 in Cooke et al. 2004a). Tags often include several sensors, alongside tracking technology (e.g., Milsar tags, include gyroscope, magnetometer, temperature, light and pressure sensors, and GPS; milsar.com/p/11-radiotag-telemetry). Some combinations are relatively standard for specific studies (e.g., time, temperature and depth recorders for fish; www.lotek.com/products/lat1000-series). A well-developed literature exists around the more common types of biotelemetry, including general (e.g., Cooke et al. 2004a, Ropert-Coudert and Wilson 2005) and specific reviews (aquatic, Hussey et al. 2015; terrestrial, Kays et al. 2015); other types of biotelemetry are more experimental. We outline below the main types of biotelemetry technologies, with example applications.

### Imagery

Thanks to camera miniaturization, animal-borne videography can obtain information about the ecology (diet, habitat use) and behavior (mate selection, threat avoidance) that could be critical for conservation. For example, video loggers have been deployed to study ecology and behavior—for example, on crows (Rutz and Troscianko 2013) and Tasmanian devils (Andersen et al. 2020). So-called AVEDs (animal-borne video and environmental data collection systems) integrate environmental sensors (e.g., audio, location, temperature, acceleration). Moll and colleagues (2007) provided a review of the evolution, use and advantages or disadvantages of AVED technology. An important limitation is that video creates large amounts of data.

### Audio

Microphones can be mounted on animals, independently or alongside a camera or other environmental sensors (often position, movement and orientation). They can record sounds produced by the animal carrying the microphone or tag, its conspecifics, but also the amount of sound an animal is exposed to. Acoustics also generates large amounts of data, and storage and manual reviewing are usually limiting factors. They have also been deployed on terrestrial and aquatic species. Johnson and colleagues (2009) reviewed the technology evolution and use for marine mammals (sound is key for their communication). Small acoustic devices have been developed recently (e.g., less than 1 gram [g] microphone backpack; Gill et al. 2016), including the recent open-source µMoth (www.openacousticdevices.info/mmoth).

### Physiology and energetics

Many physiological parameters (e.g., body temperature, heart rate, metabolism, cellular respiration, blood flow, muscle activity, neural activity) can be measured with sensors attached externally or implanted within the animal's body (Laske et al. 2018)—for example, tiny transmitters (less than 1 g) to monitor heart rate, wing beat rate, and respiration in free-flying songbirds and bats (Cooke et al. 2004a) and electromyograms to identify stressors in fish to improve knowledge of salmon migration ecology (Cooke et al. 2004b) and to measure heart rate, temperature, and activity in ruminants (Signer et al. 2010). Physiology and energetics studies are often complemented with fine-scale positional data (see the next section); this combination could help prevent poaching (Laske et al. 2018).

### Body position and movement

Body position (e.g., head down could indicate grazing) or the amount of movement (e.g., acceleration achieved or speed profiles) is sometimes of interest when the focus is on behavior and interaction with the immediate environment, rather than absolute position or movement over the landscape (as in wildlife tracking). Accelerometers can provide high-resolution data to estimate energy expenditure and activity budgets (Brown et al. 2013), detect rare behavioral events such as predation (Rutz and Hays 2009) and derive behavioral states from body position (Martiskainen et al. 2009). Pressure sensors can monitor depth within the water column (e.g., cetacean diving profiles) or altitude of a bird during migration (Shipley et al. 2018). Magnetic contact switches can log events triggered by movement (e.g., prey ingestion by recording jaw opening; Plötz et al. 2002). Hall sensors can track the movement of body parts with small magnets attached to them (Wilson and Liebsch 2003).

### Environmental sensing

Biologging tags can also collect high-quality data on the environment in which animals live, with two distinct purposes: studying how these animals relate to their environment, or using them as vehicles for environmental data collection. For example, marine animals are being used as oceanographers (Rutz and Hays 2009), with, for example, deep-diving elephant seals with conductivity, temperature, or depth sensors have become an essential source of temperature and salinity profiles in polar oceans (eurogoos.eu/download/IN-SITU-OBSERVATIONS-USING-TAGGED-ANIMALS-March-2017.pdf).

### Individual identity: RFID and PIT tags

Identifying individual animals is important for many conservation studies and statistical methods (e.g., mark–recapture). Although low-tech marking options exist (e.g., visible and fluorescent tags; Catalano et al. 2001), technologies such as RFID and PIT tags can provide a robust long-term way to obtain unique identity for individuals, that in some cases can enable automated identification. Radio-frequency identification (RFID) uses an electromagnetic field to identify tags at (typically) short distances, and recover very simple information (e.g., individual identity code). RFID tags can be active (battery-supported constant transmission), battery-assisted passive (activated by a reader, but detection over longer distances thanks to a battery), or passive (no battery)—see Baratchi and colleagues (2013, section 4.1) for more details. The transponders of passive RFID systems (PIT tags, for *passive integrated transponders*) are the cheapest and smallest but require harvesting the electromagnetic field of the reader to transmit the information (e.g., identity) encoded in them, so they typically work at close or very close (touching) range. RFID has become extremely common in industry, commerce and agriculture. Commercial readers (approximately hundreds to thousands of dollars), much more expensive than tags (from a few cents), have historically limited the use of RFID in ecological studies. They have become increasingly accessible over the last decade, and even low-cost DIY alternatives have been designed for wildlife monitoring (e.g., open-source reader; Bridge and Bonter 2011).

PIT tags have long been used in wildlife studies, either injected into the animal's body or attached to it (Gibbons and Andrews 2004), and the tiny size of some tags even permits use on invertebrates (e.g., 9-microgram tags to study ant behavior; Robinson et al. 2009). PIT tags are often used for mark–recapture studies of large cohorts of fish (e.g., Zabel et al. 2005 marked more than 150,000 salmon). Demographic parameters and movement patterns can also be estimated with individual identification (Gibbons and Andrews 2004). Individual identity may be broadcasted, to locate specific individuals in large landscapes (e.g., for translocation) or read at close range (e.g., to confirm identity of illegally removed individuals; Gibbons and Andrews 2004).

### Interactions and proximity

A special use of individual identity coupled with location data is to study proximity to another sensor using proximity loggers. In the present article, the interest is in the relative position of an animal with respect to another sensor-carrying animal or to a fixed sensor (e.g., on a fence or den entrance), rather than in absolute position. The proximity event is most often detected locally. For example, some proximity tags transmit an identifying signal and log duration of each proximity event—that is, when another tag comes closer than a predefined distance (Drewe et al. 2012). Proximity can also be derived externally using tracking data (e.g., GPS fixes; Böhm et al. 2008). Animal-borne proximity loggers tend to be expensive and often bulky, but smaller loggers have been developed recently (e.g., Encounternet system; Mennill et al. 2012). Proximity between two animals has been used to study intra- and interspecies interactions (see references in Drewe et al. 2012). Proximity to a fixed detector can also be ascertained with RFID (for reviews, see Gibbons and Andrews 2004, Bonter and Bridge 2011). Potential conservation applications include tracking the use of specific features (e.g., caves, culverts under highways, bird feeders and nest boxes).

## Wildlife tracking

Wildlife tracking (position telemetry) is one of the most mature areas of conservation technology. The focus is on determining the absolute position (coordinates) of individuals and their movement over time (and, sometimes, associated quantities such as speed and acceleration). Real-time position knowledge can help locate a specific individual (e.g., for recapture) and assist wildlife management (see the “Virtual fencing” section). Movement data can inform about behavior and habitat preferences, supporting management (e.g., habitat protection or restoration priorities, the use of road underpasses). Tracking data, biotelemetry and remotely sensed habitat data together can provide a high-resolution picture of how and when animals move and interact with each other. There is a considerable literature around wildlife tracking, including general advice on deployment and data collection (e.g., manuals; Pride and Swift 1992, Kenward 2000); statistical modeling (Patterson et al. 2008); review and future developments (Bridge et al. 2011, Baratchi et al. 2013, Hussey et al. 2015, Kays et al. 2015); technology recommendations based on animal weight, environmental limitations, and technical requirements (Thomas et al. 2012). The basic trade-offs are between size, price and data collection capacity. There are two distinct flavors of studying movement ecology on the basis of spatiotemporal data (Baratchi et al. 2013): individual based (tracking some tagged individuals) versus place based (gathering detections at fixed detectors and fitting models that estimate probability of presence of an individual at a location); most technologies described in the present article belong to the first category. For example, bird migration can be studied by tracking a few individuals or by counting passing birds at fixed stations.

### Technology overview

Most tracking systems involve at least one emitter and one receiver, one of which is animal borne. Tracking technologies can be divided in two main groups, depending on whether an animal-mounted device (tag) receives an external signal that allows it to calculate its coordinates locally (e.g., by triangulation, combining the estimated bearing to several sources) or transmits a signal that can be used externally to calculate its position, by triangulation (combining the estimated bearing to the source from several positions) or by proximity to a point of known position. Data gathered by tracking tags may be stored on board for manual retrieval, but some tags allow wireless data retrieval over relatively short distances (using VHF or UHF—ultrahigh frequency—receivers). Automated data downloading is possible, via satellite-based systems (e.g., Argos, Iridium, Globalstar), the cellular network (e.g., GSM) or, in some cases, WSNs. See (Bridge et al. 2011, Thomas et al. 2012) for more details.

Many telemetry devices (e.g., radio collars, fish tags) are produced by a few companies specifically developing technology for wildlife management, research, hunting or agriculture or aquaculture. The recent OpenCollar initiative (opencollar.io) aims to develop open-source tracking collar hardware and software, potentially bringing down costs and providing higher design flexibility. Except in fisheries and a few terrestrial exceptions, tracking studies often involve a handful of individuals. Several initiatives pull data together from many individual studies. Movebank (www.movebank.org) is a free online database and web application for archiving and sharing wildlife tracking data (Kranstauber et al. 2011). Other national and international collaborations, often taxon specific, include the Ocean Tracking Network (oceantrackingnetwork.org), IMOS (the Integrated Marine Observing System; imos.org.au) and EUROMAMMALS (euromammals.org).

### Radio-frequency triangulation

From its origins in the 1960s (Cochran and Lord 1963), radio-frequency-based tracking become a standard for wildlife tracking, only challenged by the emergence of satellite-based tracking in the 1990s, which massively improved area coverage. Radio-tracking collars emit a radio signal (commonly VHF, sometimes UHF) that can be tracked using a directional antenna. Each fix only provides the bearing to the tag, but a location estimate can be derived by triangulation by taking successive measures from different positions. Although radio-tracking collars were initially big (battery weight still often the limiting factor), technology miniaturization currently allows tagging small species (e.g., insects; Kissling et al. 2013). VHF tracking is labor intensive (it might take a day to locate an animal) and has limited coverage (a few hundred meters around smaller tags). Drone-based VHF triangulation has been trialed to avoid the difficulties of manual radio tracking (Cliff et al. 2018, Shafer et al. 2019). Automated triangulation can be performed with fixed receivers (e.g., Kays et al. 2011).

### Global navigation satellite system

Global navigation satellite systems (GNSS) are satellite-based navigation systems with global coverage. GPS (the US Global Positioning System) was the first (and most popular) system, but others have been developed (Russia's GLONASS, China's BeiDou Navigation Satellite System, EU's Galileo). A constellation of satellites orbiting the Earth continuously transmit radiofrequency signals that can be read by GPS tags; by calculating distances to several satellites of known position, the GPS tag can estimate its position. Location fixes can be stored on board or retrieved wirelessly. Cagnacci and colleagues (2010) and other above-mentioned reviews discuss the applications and potential of GPS-based wildlife telemetry. Although historically tag and battery size (more than 10 g; approximately 30 g with data transmission) limited the use of GPS to larger animals, modern lightweight GPS tags (2.5 g) have been deployed on medium-to-small size animals for extended periods (Recio et al. 2011). Fastloc-GPS (a proprietary improvement for rapid satellite signal acquisition; wildlifecomputers.com/data/technologies/fastloc) allows practical deployment of GPS tags for marine animals that only surface for a few seconds (Dujon et al. 2014). High tag cost (more than $2000) often limits the number of tracked individuals. Low-cost DIY GPS collars have been developed (e.g., with off-the-shelf GPS receivers, Allan et al. 2013; based on microcontrollers, Foley and Sillero‐Zubiri 2020) but are not widely used.

### Satellite-based tracking

In contrast to the tags of GNSS systems, which calculate position locally from received signals, the tags of the long-standing Argos System(www.argos-system.org) periodically transmit a radio signal to a suite of polar-orbiting satellites, which relay it to ground stations where location is calculated (on the basis of the Doppler effect due to the satellite's speed). It provides near-real-time global coverage, although with much coarser location accuracy (often more than 100 m) than GPS. It can also relay data from other tags, including GPS. Traditionally limited to medium or large species, recent developments (2 g solar-powered tags; www.microwavetelemetry.com/solar_2g_ptt) allow studying movement in small birds (Kok et al. 2020).

A recent exciting development is the Icarus Initiative (for *International Cooperation for Animal Research Using Space*; www.icarus.mpg.de/en), which promises to track small animals and study migrations at a global scale (Wikelski et al. 2007) thanks to solar-powered tags weighing 5g (with plans to reach 1g, allowing deployment even on large insects). Current tags include GPS, 3D accelerometer, magnetometer, temperature and other sensors. Its central system was installed in 2019 onboard the International Space Station, flying overhead at least once a day over 90% of the Earth's surface. Hibernating tags wake up to transmit locally stored position and sensor data, which are relayed from the ISS to a ground station and stored in a central online database (Movebank) for user retrieval.

### Inertial sensors and dead reckoning

Data from accelerometers, gyroscopes, magnetometers and pressure sensors can be used to derive (2D or 3D) speed and acceleration, providing a relative measure of movement or, if coupled with an initial absolute position, a high-resolution tracking method (aka dead reckoning or inertial measurement). As position error accumulates over time, it requires regular correction from an absolute measurement (e.g., GPS). This approach can augment other methods such as GPS tracking during periods when the main system may not work (e.g., surfacing marine mammals; Johnson et al. 2009).

### Harmonic radar

Harmonic radar can track individual animals carrying small simple passive tags that reradiate an emitted radar signal (Kissling et al. 2013). Since the experimental trials in the 1990s (Riley et al. 1996), it is now an established approach to tracking insects (e.g., Cant et al. 2005) and frogs (Roznik and Alford 2015), with two main variants: Stationary scanning radar stations are more expensive but record direction and distance to the tag, up to 1 km, whereas cheaper, lightweight, handheld receivers (harmonic direction finders; recco.com) only provide bearing, which can be used for triangulation at shorter ranges.

### Acoustic triangulation

Triangulation of sound sources requires specific hardware—for example, time synchronized microphone arrays, with location estimated on the basis of differences in arrival times or amplitudes (Rhinehart et al. 2020). It can aid abundance estimation and help locate threats such as gunshots (Stevenson et al. 2015). Sound (audible or ultrasounds) is the most common form of underwater tracking because pressure waves propagate through water much better than radio waves. Sound-emitting acoustic tags have been deployed to track a variety of aquatic species, from fish (common in fisheries research) to marine mammals (Johnson et al. 2009). Acoustic triangulation is less common on land, often relying on naturally produced sounds rather than tags, which simplifies deployment but complicates analysis (sound events must be matched across receivers).

### Light-derived position

Light-level tags (*geolocators* or *geologgers*) measure light levels at regular times. Locally stored data can be retrieved and used to estimate location by inferring solar position with respect to the horizon (estimating sunrise and sunset times). This system can have poor accuracy (errors up to 200 km, particularly in latitude) and suffers from different sources of error (e.g., shading of the tag), but with tag weights down to 0.6 g, it is often the only option to track small birds (study migration routes, phenology, overwintering areas) at global scale. Bridge and colleagues (2013) review the use of geolocators on small birds, and Lisovski and colleagues (2020) explain the concepts and provide a practical guide to the curation and analysis of geolocator data.

### Fixed detection stations

The alternative to tracking the movement of tagged individuals is for fixed stations (sometimes organized in large networks) to detect the proximity of marked individuals. Traditional mark–recapture studies (e.g., banded birds detected at fixed stations) fall in this category, but in the last decades, technology has offered automated alternatives. Although some sensor types described in earlier sections (e.g., camera traps) can identify individuals on the basis of variation in their natural markings, some systems have been developed specifically for tracking. For example, the Australian IMOS (imos.org.au) includes small acoustic tags attached or implanted on marine species (crustaceans, fish, marine reptiles and mammals) by different research groups, which transmit unique identifier codes. Arrays of receivers around the country detect tags of passing animals and read their identity, automatically providing information about movement patterns. Similarly, the Motus Wildlife Tracking System (motus.org; Taylor et al. 2017) creates a network of land-based telemetry stations to track small flying animals, currently spanning 31 countries and more than 200 species tagged.

PIT tags (see the “Individual identity” section, under “Animal-borne devices”) can also be used in movement studies, on the basis of fixed stations with automated RFID scanning (Smyth and Nebel 2013). Given the short range of PIT tags, detectors are often located, for example, at large concentrations (e.g., colonies) or movement bottlenecks (e.g., road underpasses or culverts).

We end this section covering two types of wildlife tracking applications, which are likely to become increasingly used.

### Virtual fencing

Virtual fencing is the use of nonphysical barriers or virtual boundaries, to control the location and movement of animals. Conservation applications are still relatively few but increasing (reviewed by Jachowski et al. 2014). It avoids blocking movement of nontarget wildlife species and people, and is cheaper than physical fences. Tracking technology allows two options. Animal-mounted training collars (livestock, www.agersens.com/eshepherd; within wildlife, mainly wolves to date) act as proximity alarms, either triggering on-the-ground alarms (visual or auditory radio-activated guards) or delivering a cue to the animal (e.g., electric shock or sound) when they cross a virtual boundary. Real-time virtual fencing (geofencing) use animal-mounted real-time position tracking (e.g., GPS) to track position compared with some virtual fences (e.g., edge of wildlife reserve or farmland areas), with a notification sent to conservation staff or private landowners when an animal crosses a virtual fence, triggering an appropriate management response (e.g., translocation). Virtual fencing has been implemented to reduce human–wildlife conflict (box 1 in Jachowski et al. 2014, Anastácio et al. 2018) and to protect critically endangered species (e.g., avoid Californian condors colliding with wind turbines; Sheppard et al. 2015). Animals can also be detected using fixed sensors (e.g., the Vertebrate Pest Detect-and-Deter; www.csiro.au/en/News/News-releases/2017/Keeping-pests-at-bay-the-hi-tech-way).

### Large-scale integrated surveillance systems

Data from different animal-borne sensors (including tracking) can be integrated in large-scale surveillance or monitoring systems. For example, Wall and colleagues (2014) developed a real-time monitoring system for elephants on the basis of GPS fixes transmitted to a cloud-based control system. Analysis of position data (e.g., for geofencing) and movement–behavior data (e.g., movement rate or immobility can indicate injury or death) allows rapid intervention. Similarly, the Domain Awareness System (www.vulcan.com/News/2017/Domain-Awareness-System.aspx) integrates data from several technologies in protected areas in Africa to provide real-time information on biodiversity, assets (e.g., patrols) and threats, to improve management and ranger deployment.

## Actuators

Actuators, the parts of a device that move and control a mechanism or system (e.g., gates, motors, pumps), are used to react on sensor information (Aqeel-ur-Rehman et al. 2014). Although not a new idea, conservation applications are still uncommon and limited to some specific situations that require acting on a sensed cue. We present in the present article a few examples and ideas, some at development and testing phases.

Wildlife traps may use electronics (e.g., motion sensors) to trigger the trap (Kittelson 2016); more advanced approaches could include individual (e.g., PIT tag reader) or species identification (e.g., from a camera) for targeted trapping. Some (e.g., for feral pigs) can also be activated remotely (www.buckeyecam.com/site/assets/x80_activator_product_brief.pdf). Bait stations could have actuators that deliver toxins for eradication (e.g., possums in New Zealand). Grooming traps are being trialed to spray a toxin on feral cats or foxes in Australia as they walk in front or inside a device, with infrared beams used as triggering mechanism (Read et al. 2014). Actuators can be used in remote feeders to release food in a programmed way (e.g., Parrott and Chasse 2005) or in response to local cues or remote monitoring through wireless camera traps (e.g., BuckEye Cams; www.buckeyecameras.com/products.html). Actuators have been used as deterrents to reduce human–wildlife conflict and animal–vehicle collisions (see the “Virtual fencing” section above).

## Computing

If the previous technologies represented the body (physical objects, or *hardware* in electronics lingo), we now turn to the brains, the more abstract functions of storing, manipulating and analyzing data, and making automated decisions. This includes algorithms (sets of rules that define a sequence of operations), signal processing and statistical methods (abstract ideas). These are implemented as software (programs or applications), which run on some piece of hardware with computing capabilities (e.g., a computer or microcontroller). Recent advances in and availability of computing and processing algorithms is expected to help realize the full potential of other technologies (e.g., data-gathering sensors). This section covers some selected computing technologies and applications that are having a substantial impact in conservation and biodiversity research. We do not discuss the most basic computing functionalities (e.g., spreadsheets, databases, GIS—geographic information system mapping) not specific to conservation.

### Websites and online platforms

Databases or data repositories have become powerful tools thanks to online access through the Internet, from any connected device, including computers, mobile phones and tablets, but also increasingly automated devices without human supervision. Remote access is important both ways: Data can be retrieved at any time, but can also be submitted to online databases, manually (by humans) or automatically (by connected sensors). Classic sources of biodiversity records (museum or herbarium collections, dedicated sampling) are currently augmented with other sources, including DNA sampling, citizen science and remotely sensed data (Pimm et al. 2015). The front end (i.e., what the user sees) of online databases or platforms is often a website (a portal) or smartphone app, which facilitates manual data entry.

The power of these technologies resides in facilitating a massive accumulation of data over geographic and temporal scales that would be otherwise impossible. Some global online platforms such as the GBIF (Global Biodiversity Information Facility; www.gbif.org) or eBird (www.ebird.org) gather millions of records. Many other international, national (e.g., Atlas of Living Australia; www.ala.org.au) and even regional data portals exist, some of them with specific taxonomic focus (see examples in August et al. 2015). Some deal with specific data types (e.g., Xeno-canto for acoustics, www.xeno-canto.org; Movebank for animal tracking data, www.movebank.org); others have specific functionalities (e.g., iNaturalist, an online social network of citizen scientists and biologists that share observations; www.inaturalist.org).

### Mobile phones and apps

Mobile phones (including smartphones), ubiquitous across the world, represent one of the most powerful technology advancements for conservation. They can provide Internet access and allow easy deployment of applications (apps, small task-oriented programs) to facilitate a variety of objectives, including data entry by professionals, data gathering in citizen science or community monitoring projects, society and community engagement, and education. For example, the Forest Watcher app (forestwatcher.globalforestwatch.org) can be used to track and document deforestation (e.g., illegal logging events). Many repositories mentioned in the previous section (e.g., eBird) have associated mobile apps to facilitate data recording (with associated metadata; e.g., GPS coordinates). Bespoke data collection apps can be easily created or customized (e.g., Cybertracker for GPS and auxiliary data, www.cybertracker.org; the highly customizable open-source community-driven app development platform Open Data Kit, opendatakit.org). Low cost allows bottom-up citizen science to gather data and address local issues, avoiding the traditional reliance on institutions (August et al. 2015). Importantly, user interface and user experience must consider the target audience, which may include illiterate users or speakers of minority languages (e.g., pictograms in Cybertracker).

### Crowdsourcing

Citizen science (the contribution of nonscientists to gathering data of scientific value) represents a form of crowdsourcing (outsourcing work to the crowd), where many individuals contribute to a common goal. Some of the largest citizen science communities include Zooniverse (www.zooniverse.org), iNaturalist and eBird. Through the Zooniverse web platform, individuals can help gather data for scientist-driven projects. Crowdsourcing can also harness the pattern recognition ability of the human brain, using volunteers to identify animal species in camera trap pictures accessed through an online portal or mobile app (e.g., Instant Wild; instantwild.zsl.org/intro). New technologies will bring opportunities and challenges to realize the potential of citizen science (Newman et al. 2012). Crowdfunding, another form of crowdsourcing, relies on online platforms such as Kickstarter (www.kickstarter.com) to allow people around the world to contribute financially to conservation projects (Gallo-Cajiao et al. 2018).

### Artificial intelligence: Automation and autonomy

Artificial intelligence (AI) is one of the big technology promises for the coming decades, particularly coupled with big data. AI is a broad term that includes machine learning methods for data analysis (computational alternatives to classical statistics). We do not include in the present article classic forms of data analysis because they form a distinct body of knowledge not often associated with the term *technology*. Some of the algorithms required for AI fall within the discipline of signal processing. AI promises to bring automation (of tasks traditionally done by humans) and autonomy (facilitating or even taking over the process of decision-making).

Automation based on AI algorithms could strongly benefit conservation by accelerating the extraction of useful information from the increasing amounts of data being collected (Kwok 2019), particularly by sensors such as camera traps and acoustic loggers; such time-consuming tasks have traditionally been performed manually, often becoming the limiting factor of a project. Automation could even provide insights in near real time. The strong industry push for AI-based automation is also starting to benefit conservation (e.g., Microsoft's AI for Earth grant program; www.microsoft.com/en-us/ai/ai-for-earth-grants). Deep learning, a family of artificial neural network approaches (LeCun et al. 2015), has recently become notorious for their success at achieving complex identification tasks. Their spread is being facilitated by open-source platforms supported by industry giants (e.g., Microsoft Cognitive Toolkit version 2.0, Google's Tensor Flow library). A recent review describes existing uses of deep learning in ecology (Christin et al. 2019).

A popular application is in automated identification of species (or sometimes even individuals) from content-rich data types such as images (including thermal; Corcoran et al. 2019), video and audio. It typically requires specialized skills to train models, but programs exist to facilitate this task. Recent examples with impressive performance include species identification from camera trap images (Norouzzadeh et al. 2018); the cloud-based Wildbook (wildbook.org), which can identify and track individuals of most striped, spotted, wrinkled or notched species; iNaturalist's SEEK app (www.inaturalist.org/blog/23075-real-time-computer-vision-predictions-in-seek-by-inaturalist-version-2-0) for real-time species identification with smartphone cameras. Some applications target specific taxa (e.g., salamanders; Gamble et al. 2008) or may be broad (e.g., electronic surveillance of counting and sizing fish in fishing vessels to help protect fisheries; civileats.com/2018/05/10/the-future-of-fish-is-big-data-and-artificial-intelligence). Automated identification of humans in camera traps can help combat poaching (e.g., TrailGuard AI camera trap sends immediate wireless alerts to parks management when a person is detected, www.resolve.ngo/trailguard.htm; trials in Tanzania led to 30 arrested poachers). Automated species identification within sound recordings has also advanced to the point where relatively high performance can be expected for many species (Stowell et al. 2019). Other automated tasks include automated counting of individuals of a target species from remotely sensed images, including from satellites (e.g., savannah mammals; Yang et al. 2014) and drones (e.g., seabirds in large colonies; Hodgson et al. 2018) and automated tracking of a moving object in video footage (Dell et al. 2016).

Autonomy is a growing area of application of AI algorithms. Real-time species identification is key to automating decision-making. For example, bait dispensers could release poison bait only when the target pest species is captured (grooming traps trialed for culling feral cats; www.ecologicalhorizons.com/assets/feral-cat-grooming-trap-jan2015.pdf). AI algorithms were traditionally trained and applied on local powerful computers or in the cloud (large dedicated computing facilities accessed online) but the processing power of higher-end smartphones currently allow some AI applications to run locally (www.inaturalist.org/blog/23075-real-time-computer-vision-predictions-in-seek-by-inaturalist-version-2-0). Furthermore, recently developed dedicated hardware (e.g., Google's Coral microcontroller, coral.withgoogle.com/docs/edgetpu/models-intro) for so-called edge AI or edge computing (www.imagimob.com/blog/what-is-edge-ai) allows algorithms (still trained on powerful computers) to be deployed and used in real time even on relatively simple devices (e.g., sensors, such as a camera trap or actuators), with potential to provide local decision-making capabilities. Autonomous navigation, underpinning (e.g., self-driving cars and autonomous drones) could have useful applications for conservation. Experimental examples already exist (e.g., RangerBot AUV, programmed to detect and kill invasive crown-of-thorns starfish in the Great Barrier Reef; www.wildlabs.net/resources/news/underwater-robot-trained-kill-coral-destorying-reef-starfish).

### Low-cost computing

The last 10 years or so have witnessed a revolution in low-cost computing, with several boards based on simple processors or microcontrollers (“dumber” cheaper version of the microprocessor that run the brains of a computer) have been developed commercially that are much simpler to use—even without specialized skills (Cressey 2017). Single-board processors such as Raspberry Pi (www.raspberrypi.org) and microcontroller-based boards such as Arduino (www.arduino.cc) have been heralded as opening the world of electronics to the general public. It has revolutionized the world of DIY electronics and the maker community with extremely low prices (e.g., approximately US$4 for Arduino), online communities (e.g., Instructables, www.instructables.com) providing discussion and support and (often free) learning materials. Low-cost computing coupled with DIY electronics has been advocated as key to revolutionize wildlife data gathering, particularly in the context of the open-source movement (Greenville and Emery 2016). Recent examples include a Raspberry Pi–based open-source acoustic platform (Whytock and Christie 2016) and an Arduino-based radio-tracking collar (Foley and Sillero‐Zubiri 2020). Low-cost computing is not limited to these easy-to-use commercial boards. Others have integrated more efficient (e.g., better use of battery) microcontrollers into conservation-oriented products (e.g., AudioMoth acoustic device, Hill et al. 2018), but note this approach requires specialized engineering skills.

## Conclusions

Our overview showcases the stunning breath of applications of technology in conservation. There has been a long tradition of using technology to aid studies of wildlife, reflected in some well-established tools, including camera trapping and radio tracking. But recent decades have seen a dramatic escalation in technology use and sophistication, particularly thanks to increased availability of remotely sensed products, computing power, and cheaper electronics. We conclude by synthesizing some key observations and trends.

### From monitoring and research to conservation action

Many conservation technologies are used for wildlife and habitat monitoring. Data gathered using these devices and systems are essential to many conservation studies and, perhaps more importantly, underpin solid decision-making in conservation management. Often the purpose of monitoring is to ascertain the presence of a species or to track changes in species distribution and abundance (e.g., through capturing images or sounds). More complex data types (e.g., related to animal movement, behavior or physiology) are typically used in research to gain insight that can then be applied to aid conservation work. Fewer technologies are specifically created for on-the-ground conservation action (e.g., drones for antipoaching patrols, satellite imagery to detect deforestation in protected areas, virtual fencing, automated baiting stations).

### From well-established to more recent applications of technologies

Conservation technologies can be classified on a continuous spectrum from well-established technologies that have become standard tools in conservation research and projects (e.g., radio tracking, camera traps), to novel applications of technology that are still not in widespread use (e.g., drone-based radio tracking, deep learning algorithms for automated detection of sounds or images). We can expect many of these novel applications to become established tools within the next decade. We note that *well established* should not be taken to mean outdated or underperforming; on the contrary, these technologies have often continued evolving following industry and research developments (e.g., intelligent camera traps, GPS-based wildlife tracking compared with earlier VHF tracking).

### From generic to specific technologies

To date, conservation has mostly used technologies developed for other purposes—for example, military, consumer market or biomedical (Berger-Tal and Lahoz-Monfort 2018). Targeted development for conservation is increasingly more common as one progresses along the technology pipeline (figure 1): Very few (if any) sensors are created specifically for conservation; some devices are developed for biodiversity-related purposes (e.g., radio-tracking collars, acoustic loggers), but many others are not (e.g., thermal scopes, radar stations, AI methods); most systems established for conservation or ecology purposes are specifically built (even if using generic devices and sensors as elements); many if not most applications, web-based services, or algorithms (e.g., automatically identifying species in images, web portal for crowdsourcing) are specific to their conservation use (although run on computing resources and knowledge that are generic).

We believe this is likely to change in the near future, with calls to support the development of more targeted conservation technology (Lahoz-Monfort et al. 2019), especially at the device level. Whether generic or specific to conservation or ecology, most devices are commercial products; some open-source or DIY options exist, but they are currently the exception (we have highlighted some of these options above). This situation can be expected to change over the next decade (see below).

### From labor intensive to increased automation

Early use of technologies in conservation has often been very manual and labor intensive (e.g., manual VHF radio tracking, walked transects with handheld thermal scopes, manual checking of images from camera traps or sounds from acoustic monitoring). However, changes over the last decade in many aspects of the technology pipeline have allowed an increasing volume of data being collected (cheaper technology), processed (cheaper computing), made widely available (Internet connectivity) and analyzed (statistical methods, automated analysis of images and sound, crowdsourcing tasks). Raw data are often only the first step, and can become a problem if they accumulate faster than they can be dealt with. Even now, there is often a substantial gap, with cheaper and more available devices creating volumes of data (particularly image and sound) that are difficult to handle and analyze. The bottleneck in biodiversity monitoring technology is moving from data acquisition to data handling. Advances in AI methods are enabling a much-needed increase in automation. This is a key development and we expect the situation to improve in the coming decade.

### The next decade of conservation technology

We believe the following (in no particular order) are promising avenues for technology to aid conservation in the near future: AI-based automation and autonomy; increased integration of different technologies and associated data (Marvin et al. 2016); the Internet of Things providing increased capacity for sensors to talk to each other; open-source innovation providing new ways of designing, prototyping, and manufacturing technology specifically for conservation purposes (Lahoz-Monfort et al. 2019). Although outside the scope of our overview, we close this list mentioning another promising area (Corlett 2017): genetics or molecular technology providing increasing capacity to detect, identify, and manage populations and species and to modify organisms for conservation and environmental purposes (e.g., gene drive introducing disease resistance or sterility in invasive species).

We argue that the success of conservation technology as an emerging discipline will depend on both sides: developers and users. On one hand, learning how best to attract technologists to collaborations with the conservation community and ensure the technology prototyped can be converted into final products that can be scaled up for global impact and remain viable over the longer term (Lahoz-Monfort et al. 2019). On the other hand, we need to ensure that technology is fit-for-purpose (including withstanding harsh field conditions) and appropriate for the socioeconomic and cultural context in which it will be used (e.g., avoiding creating new forms of dependency of biodiversity-rich developing countries on developed nations). Open-source innovation may be a way forward to achieve these objectives.

The road ahead for conservation technology looks promising but challenging as the conservation community learns to better collaborate with technologists and avoid the pitfalls of misguided use. Our overview highlights that technology can make a great contribution to the conservation toolkit, but in the end, conservation primarily deals with human societies and human behavior, with all their complexities. Only by mastering all these aspects, and not simply developing new cool tech, will conservation technology achieve global impact to aid conservation in the twenty-first century.

## Author Biographical

José J. Lahoz-Monfort (jose.lahoz@unimelb.edu.au) is affiliated with the School of Ecosystem and Forest Sciences at the University of Melbourne, in Melbourne, Victoria, Australia. Michael J. L. Magrath (mmagrath@zoo.org.au) is affiliated with Wildlife Conservation and Science at Zoos Victoria and with the School of BioSciences at the University of Melbourne, in Melbourne, Victoria, Australia.
